# Acute oxytocin effects in inferring others’ beliefs and social emotions in people at clinical high risk for psychosis

**DOI:** 10.1038/s41398-020-00885-4

**Published:** 2020-06-22

**Authors:** André Schmidt, Cathy Davies, Yannis Paloyelis, Nicholas Meyer, Andrea De Micheli, Valentina Ramella-Cravaro, Umberto Provenzani, Yuta Aoki, Grazia Rutigliano, Marco Cappucciati, Dominic Oliver, Silvia Murguia, Fernando Zelaya, Paul Allen, Sukhi Shergill, Paul Morrison, Steve Williams, David Taylor, Stefan Borgwardt, Hidenori Yamasue, Philip McGuire, Paolo Fusar-Poli

**Affiliations:** 1 Department of Psychiatry (UPK), University of Basel, Basel, Switzerland; 2 Early Psychosis: Interventions and Clinical-detection (EPIC) lab, Department of Psychosis Studies, Institute of Psychiatry, Psychology & Neuroscience, King’s College London, London, UK; 3 Department of Neuroimaging, Institute of Psychiatry, Psychology & Neuroscience, King’s College London, London, UK; 4 Department of Psychosis Studies, Institute of Psychiatry, Psychology & Neuroscience, King’s College London, London, UK; 5 National Institute for Health Research (NIHR) Biomedical Research Centre (BRC), South London and Maudsley NHS Foundation Trust, London, UK; 6 Department of Brain and Behavioural Sciences, University of Pavia, Pavia, Italy; 7 Department of Psychiatry, Hamamatsu University School of Medicine, Shizuoka, Japan; 8 Medical Institute of Developmental Disabilities Research, Showa University, Tokyo, Japan; 9 Department of Psychosocial Medicine, National Center for Child Health and Development, Tokyo, Japan; 10 Tower Hamlets Early Detection Service (THEDS), East London NHS Foundation Trust, London, UK; 11 Department of Psychology, University of Roehampton, London, UK; 12 Institute of Pharmaceutical Science, King’s College London, London, UK; 13 Department of Psychiatry and Psychotherapy, University of Lübeck, Lübeck, Germany; 14 Outreach and Support in South London (OASIS) Service, South London and Maudsley NHS Foundation Trust, London, UK

**Keywords:** Schizophrenia, Physiology

## Abstract

Social deficits are key hallmarks of the Clinical High Risk for Psychosis (CHR-P) state and of established psychotic disorders, and contribute to impaired social functioning, indicating a potential target for interventions. However, current treatments do not significantly ameliorate social impairments in CHR-P individuals. Given its critical role in social behaviour and cognition, the oxytocinergic (OT) system is a promising target for novel interventions in CHR-P subjects. In a double-blind, placebo-controlled, crossover design, 30 CHR-P males were studied using functional magnetic resonance imaging (fMRI) on two occasions, once after 40IU self-administered intranasal OT and once after placebo. A modified version of the Sally-Anne task was used to assess brain activation during inferring others’ beliefs and social emotions. The Reading the Mind in the Eyes Test was acquired prior to the first scan to test whether OT effects were moderated by baseline social-emotional abilities. OT did not modulate behavioural performances but reduced activation in the bilateral inferior frontal gyrus compared with placebo while inferring others’ social emotions. Furthermore, the relationship between brain activation and task performance after OT administration was moderated by baseline social-emotional abilities. While task accuracy during inferring others’ social emotion increased with decreasing activation in the left inferior frontal gyrus in CHR-P individuals with low social-emotional abilities, there was no such relationship in CHR-P individuals with high social-emotional abilities. Our findings may suggest that acute OT administration enhances neural efficiency in the inferior frontal gyrus during inferring others’ social emotions in those CHR-P subjects with low baseline social-emotional abilities.

## Introduction

Deficits in social functioning are core features of psychosis and predictive for the onset, development, course and outcome of this illness^1^. Social functioning impairments are a distinctive characteristic of individuals at Clinical High Risk for Psychosis (CHR-P) to the point that they represent the most impaired neurocognitive domain (Hedge’s *g* = 0.55) in this patient population^2–4^. However, currently available approaches for CHR-P individuals fail to ameliorate their social impairments, indicating the need for novel and more effective interventions that map onto core pathological processes underlying the CHR-P features^5,6^. Mind-reading, i.e., the ability to infer others’ mental states, is integral for social interactions^7^ and impaired in early psychosis patients^8^ and CHR-P subjects^9,10^. Mental state attribution or perspective taking is a complex multidimensional construct and involves the ability to infer the thoughts or beliefs of others (i.e. cognitive empathy), but also the ability to infer the emotions or feelings of others (i.e. emotional empathy)^11^. Past evidence showed that cognitive and emotional aspects of empathy are partly dissociable systems^12^. While neurocognitive dysfunction significantly accounts for impairments during theory of mind (ToM) tasks in CHR-P individuals^10^, they still remain evident after adjusting for deficits in neurocognition^8^, indicating the crucial contribution of impaired emotional empathy to ToM deficits in CHR-P. Meta-analytical evidence identified the left anterior midcingulate cortex/dorsal anterior cingulate cortex, bilateral insula/inferior frontal gyrus and bilateral supplementary motor area as being consistently activated in empathy^13^. While the bilateral anterior insula/inferior frontal gyrus is specifically activated during emotional empathy, activation in the left midcingulate cortex and left anterior insula is more related to the cognitive aspect of empathy^13^. Previous functional magnetic resonance imaging (fMRI) studies in CHR-P individuals reported increased activation in the prefrontal cortex including the inferior frontal gyrus, posterior cingulate cortex and the temporoparietal cortex during inferring others’ mental status relative to healthy controls, suggesting a compensatory overactivation of brain regions critical for empathic responses during mental state attribution^14,15^. Another study observed reduced bilateral inferior frontal gyrus activation during inferring others’ emotions but not beliefs in CHR-P subjects relative to healthy subjects^16^.

Given its critical role in human social behaviour and cognition^17^, the oxytocinergic (OT) system is a promising target for the treatment of social impairments in CHR-P subjects. OT has been proposed to be a key neural substrate that interacts with central dopamine systems^18^ and may counteract hyperdopaminergia seen in psychosis^19^. Previous studies have reported altered blood OT levels in schizophrenia patients^20,21^, which predicted social cue recognition^21^. CHR-P individuals show significantly decreased OT receptor gene methylation, which was related to increased anhedonia-asociality^22^. OT administration improves mind-reading^23^ and trust^24^ in healthy volunteers. Notably, OT affects emotional rather than cognitive empathy in healthy people^25^ and its effect on mind-reading depends on baseline social-emotional abilities^26^. A single dose of OT also improves emotion recognition^27–29^, higher-order social cognition (such as appreciation of indirect hints and recognition of social faux pas^30^), as well as working memory^31^ in schizophrenia patients. However, evidence for OT effects on cognitive and emotional empathy in early psychosis is sparse. A first study showed that 24 IU for 6 weeks did not improve cognitive empathy in individuals with early schizophrenia-spectrum psychotic illness^32^.

This study investigated, for the first time, whether acute OT administration changed the ability to successfully infer on others people’s beliefs (cognitive empathy) and social emotions (emotional empathy) in CHR-P males, and whether this change goes along with altered neural activation. Based on previous evidence in healthy people^25,33^ and CHR-P individuals^16^, we expected that OT would enhance successful recognition of others’ facial emotions but not beliefs and that this improvement in successful social emotion inferences would be accompanied by increased neural activation in the emotional empathy network, in particular in the inferior frontal gyrus. As previously shown^26,34^, we further predicted that OT effects would be moderated by baseline social-emotional abilities, with CHR-P individuals low in social-emotional abilities showing greater improvements after OT administration.

## Materials and methods

### Participants

The study received National Research Ethics Service approval (14/LO/1692) and all subjects gave written informed consent. The participants and OT administration have previously been described in our Arterial Spin Labelling (ASL)^35^ and magnetic resonance spectroscopy (^1^H-MRS)^36^ studies. Sample size was estimated using G*Power 3 to detect neurochemical effects based on a previous ^1^H-MRS study^37^, which indicated that a sample size of 30 was sufficient to detect a medium within-subject effect size (*d*_*z*_ = 0.53), when alpha=0.05 and power=80%. Thirty male, help-seeking CHR-P individuals aged 18–35 were recruited from two specialist early detection services—the OASIS^38^ and Tower Hamlets Early Detection Service (THEDS), which belongs to the Pan-London Network for Psychosis-prevention (PNP)^39^. CHR-P status was assessed using the Comprehensive Assessment of At-Risk Mental States (CAARMS) 12/2006 criteria^40^. In brief, subjects met one or more of the following subgroup criteria: (a) attenuated psychotic symptoms, (b) brief limited intermittent psychotic symptoms (BLIPS, psychotic episode lasting <1 week, remitting without treatment^41–43^), or (c) either schizotypal personality disorder or first-degree relative with psychosis^40^, all coupled with functional decline. Individuals were excluded if there was a history of previous psychotic disorder (with the exception of BLIPS, some of whom may meet Acute and Transient Psychotic Disorder criteria^42^) or manic episode, exposure to antipsychotics, neurological disorder or current substance-use disorder, estimated IQ < 70, acute intoxication on the day of scanning, and any contraindications to MRI or intranasal OT or placebo. History of Axis I disorder(s) was not an exclusion criterion due to the transdiagnostic nature of the CAARMS-based definition of the CHR-P state^44^ and the high prevalence of such diagnoses within these populations^45^. We also collected information on medication history, alcohol (Alcohol Use Disorders Identification Test), tobacco and cannabis use, functioning (Global Functioning Role and Social scales^46^) and transition status. One subject reported cannabis use prior to one scan and was therefore excluded from analysis. Furthermore, to test for potential moderation effects of baseline social-emotional abilities on OT effects^26,47^, the Reading the Mind in the Eyes Test (RMET^48^) was acquired prior to the first scan. RMET measures the ability to interpret subtle social-emotional cues from the eye region^48^. It compromised 36 photographs depicting the eye region of Caucasian posers. Stimuli were presented on a black background in the center of a computer screen. In the four corners around the picture, one target label and three distractors were simultaneously shown. Participants were asked to select the correct mental state as fast as possible. The picture remained on the screen until participants clicked on a label with the mouse. A practice trial was conducted, and a glossary was provided in paper form. Performance was determined by calculating the percentage of correct answers. Demographic and clinical characteristics of the sample are presented in Table 1.

**Table 1:** Participant demographic and clinical characteristics.

Demographic	
Age years; mean (SD)	22.48 (4.68)
Age range. years	18–34
Sex; male/female	30/0
Ethnicity (White/Black/Asian/Mixed)	15/5/3/4
Handedness. right/left	23/4
Education. years; mean (SD)	13.33 (1.9)
Clinical	
CHR-P Subtype^a^ (BLIPS/APS/GRD)	5/21/1
CAARMS attenuated positive symptoms^b^; mean (SD)	11.85 (3.44)
Baseline anxiety score^c^; mean (SD)	35.07 (9.83)
GF social score; mean (SD)	6.85 (1.46)
GF role score; mean (SD)	7.04 (1.63)
RMET^d^; mean (SD), median	25.38 (4.99), 26
Substance Use	
Current antidepressant medication (yes/no)^e^	8/19
Current antipsychotic medication (yes/no)	0/27
Current benzodiazepine medication (yes/no)	0 / 27
Current smoker (yes/no)	15/12
Cigarettes/day; mean (SD)	9.33 (6.29)
Cannabis use^f^; median (range)	2 (0–4)
Alcohol. AUDIT total; mean (SD)	6.74 (7.22)

*AUDIT* alcohol use disorders identification test, *CHR-P* clinical high risk for psychosis, *GF* global functioning (role and social) scale.

^a^Comprehensive Assessment of At-Risk Mental States (CAARMS) subgroup; *BLIPS* brief limited intermittent psychotic symptoms, *APS* attenuated psychotic symptoms, *GRD* genetic risk and deterioration.

^b^Sum of the global (severity) ratings for positive subscale items (P1-P4) of the CAARMS.

^c^Mean of pre-scan anxiety scores across conditions as measured by the State Trait Anxiety Inventory (STAI).

^d^Reading the Mind in the Eyes Test (RMET) score at the initial clinical assessment.

^e^Three subjects received Sertraline (20 mg, 25 mg and 100 mg), one Escitalopram (20 mg), one Mirtazapine (30 mg), one Amitriptyline (10 mg), one Fluexetine (40 mg) and one received 300 mg of unknown type.

^f^Cannabis use: 0 = never, 1 = experimental use (tried occasionally), 2 = occasional use (small quantities from time to time), 3 = moderate use (moderate quantities regularly/large amounts occasionally), 4 = severe use (frequently used large quantities, often to intoxication/debilitation).

### Study design

Using a randomised, double-blind, crossover design, 40 IU intranasal OT and placebo were administered in a counterbalanced fashion with a 1-week wash out period. During each challenge, subjects underwent an MRI scan which started at 11:30 h to minimise potential effects of diurnal variation in OT or vasopressin^49^. Intranasal OT administration followed recommended guidelines and a protocol adopted by a previous study conducted at our institute^49^. As done in our previous studies^35,36^, participants self-administered one puff (4 IU) of intranasal OT or matched placebo every 30 s, alternating between nostrils, until 40 IU had been administered. See refs. ^35,36^ for more details.

### Functional MRI paradigm

A modified version of the Sally-Anne task was used as previously applied in individuals with autism spectrum disorder after OT administration^50^. Participants underwent two runs consecutively, each of which lasted approximately 7.5 min. Each run consisted of 10 different stories (Supplementary Fig. 1), while every story was presented three times in a row, one for each of the three conditions: ‘control', ‘belief inference (cognitive empathy)' and ‘social emotion inference (emotional empathy)'. The identical set of 10 stories was presented during the first and second run. At the end of each story participants were required to answer ‘yes–no’. Questions were presented in a pseudorandom fashion, so that ‘yes’ questions in the first run were modified to be ‘no’ questions in the second run, and vice versa. Before scanning, all participants were shown the cartoon stories in a mock scanner and required to answer questions as in the ‘control’ condition. In this manner, participants were trained to grasp the stories without experiencing the questions of the ‘Belief’ and ‘Social emotion’ conditions.

**Fig. 1:**
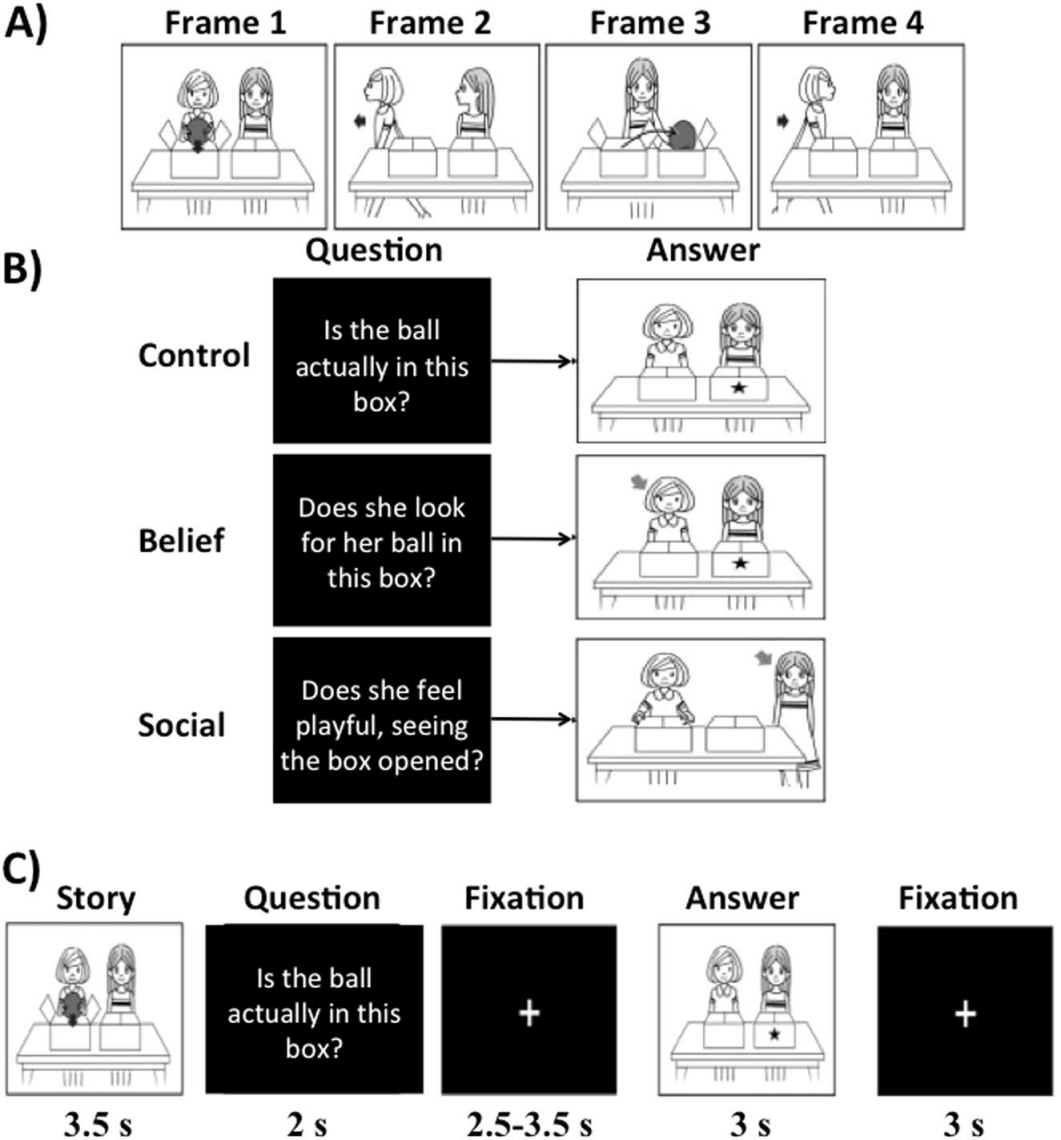
Task design. **a** Each story consisted of four frames with two people. There are 10 different types of stories (see supplementary Fig. 1). **b** Each story was presented three times consecutively per each run. At the end of each story presentation, participants were asked to answer the following three types of questions that asked emotion of the character, belief of the character, and fact (control condition). These three questions appeared in a pseudorandom order, followed by an answer frame. **c** Trial timing. 3.5 s story presentation with four frames of a black and white comic. Then, a frame with a question written in white letters on the black screen was shown for 2 s. After 2.5–3.5 s white fixation cross on the black screen, another frame was presented to illustrate an arrow and a star to help to answer the question (3 s). At the end of one story, a 3-s fixation cross was shown. During the last frame and fixation cross, participants were instructed to answer the question with yes or no.

Let us take one story as an example. As summarized in Fig. 1a, four frames show a story with two persons with neutral faces. During the story, one person (Sally) places a ball in a left box (Frame 1) and leaves the room (Frame 2). Meanwhile, another person (Anne) moves the ball to a right box (Frame 3) and then Sally returns (Frame 4). At the end of the story, participants are asked the following three types of questions in a pseudorandom order (Fig. 1b): ‘Is the ball actually in this box? (indicating a left box with a star)’ for the control condition, ‘Does she look for her ball in this box? (indicating Sally with an arrow and a left box by a star)’ for the belief inference condition, and ‘Does she feel playful, seeing the box opened?’ for the social emotion inference condition. To answer the first question (control condition), participants are required to understand the actual location of the ball. For the second question (belief inference), they are required to understand that Sally’s actions will be based on what she believes to be true, rather than the actual location. In the third question (social emotion inference), participants were required to answer the question about Anne’s emotional status after she completed the practical joke. To successfully infer on Anne’s emotional status, it is required not only to understand the story (control condition) but also that Anne believes that Sally falsely believes the ball is in the left box. In other words, successful social emotion inference (emotional empathy) depended on successful belief inference (cognitive empathy) but not vice versa. Task conditions and trial timing are summarized in Fig. 1c. Each trial consisted of 3.5 s story presentation with four frames (see Fig. 1a); 2 s question; 2.5–3.5 s fixation cross; 3 s of a frame with comic vignette that illustrates an arrow and a star to help to answer the question; and 3 s fixation cross. Participants were required to answer this question during the last frame with comic and fixation cross by pressing a button to answer yes or no.

### Behavioural analysis

We measured accuracy and reaction time to correct responses during the task. Paired t-test was used to test OT effects for each task condition.

### Image acquisition and processing

All scans were conducted on a General Electric Discovery MR750 3 Tesla system (General Electric, Chicago, USA) using a 32-channel head coil. During the task, we acquired T2*-weighted echo-planar images with the following parameters: 41 axial slices of 3 mm thickness, 0.3 mm interslice gap, field of view 24 × 24 cm^2^ and flip angle 75°. The repetition time was 2 s and the echo time 30 ms. EPIs were analysed using an event-related design with SPM12 (www.fil.ion.ucl.ac.uk/spm). Pre-processing was performed for each subject and time point separately. In brief, slice-timing correction was first performed on each volume using the middle slice as the reference to adjust for time differences due to multislice image acquisition. The images were then realigned to the first image in the series. The anatomical T1 image was co-registered with the first functional image and then spatially normalized into the MNI space, generating normalization parameters, which were applied to all functional images. Functional images were smoothed with a Gaussian kernel of 8 mm full half-width maximum (FWHM). All images were checked for movement artefacts and subjects with more than 10% corrupted volumes (all scans with more than 3 mm deviation from the previous scan in any dimension) were excluded (one subject). Voxel-wise maximum likelihood parameter estimates were calculated during the first-level analysis using the general linear model. Our design matrix included an autoregressive AR(1) model of serial correlations and a high-pass filter with a cut-off of 128 s. The onsets of each event were convolved with the SPM synthetic haemodynamic response function. Each event-related regressor had an onset at the time of the answer frame and a duration that correspond to each response time (see Fig. 1c). First-level models were constructed for both treatments separately, including only the onset times for correct responses in the control, belief and social emotion condition during both runs. In line with previous studies using the same task^16,50^, this approach allowed us to study whether OT administration changed neural activation while successfully inferring others’ belief and social emotion relative to placebo. One subject had no correct responses during one of the four runs and was therefore excluded. The final sample thus consisted of 27 subjects. Two contrast images were generated per participant: ‘belief > control’ (cognitive empathy) and ‘social emotion > control’ (emotional empathy). One-sample t tests were conducted to test for effects of condition (cognitive and emotional empathy) after placebo administration and paired t-tests were conducted to test for treatment effects in the two interrelated contrasts of interest separately as previously done^50^. Significance was assessed at a cluster-level threshold of *p* < 0.05 family-wise error (FWE) corrected across the whole brain with an uncorrected cluster-forming threshold of *p* < 0.001^51,52^ with an extent threshold of 20 voxels.

### Impact of baseline social-emotional abilities

The relationship between baseline social-emotional ability (RMET performance) and significant brain effect (left inferior frontal gyrus) after OT administration was tested using Spearman correlation at a significance level of *p* < 0.05. Parameter estimates were extracted from the whole left inferior frontal gyrus cluster (300 voxels) showing a significant effect between OT and placebo during inferring others’ social emotions (‘social emotion > control’ contrast). Nonparameteric correlation was performed due to the fact that parameter estimates of left inferior frontal gyrus activation were not normally distributed as indicated by a significant Kolmogorov–Smirnov test (*p* = 0.033). We further used a linear regression to test whether the relationship between brain activation in left inferior frontal gyrus and task accuracy after OT administration was moderated by RMET performance. Task accuracy was entered as dependent variable and the interaction RMET x left inferior frontal gyrus activation as independent variable. To perform a parametric moderator analysis, parameter estimates in the left inferior frontal gyrus after OT administration were first log transformed.

## Results

### Behavioural task performance

**Fig. 2:**
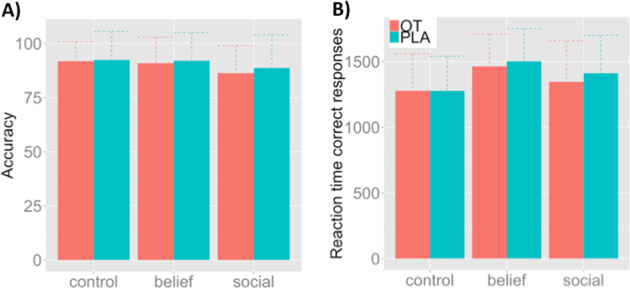
Behavioral task performance. Accuracies and reaction times in response to correct responses for both treatments during the task.

OT did not modulate accuracy for the control (*T* = 0.332, *p* = 0.743), belief (*T* = 0.618, *p* = 0.542) or social (*T* = 0.775, *p* = 0.445) condition (Fig. 2a). OT also did not influence reaction times for correct responses during the control (*T* = 0.024, *p* = 0.981), belief (*T* = 0.801, *p* = 0.430) or social (*T* = 1.11, *p* = 0.277) condition (Fig. 2b). Correlations between reaction time and accuracy within and between the different task conditions are presented in Supplementary Table 1.

### Brain activation

#### Effect of conditions

Inferring others’ beliefs (belief > control) evoked significant activation in left precuneus, angular gyrus, inferior frontal and parietal gyrus and bilateral middle frontal gyrus (Supplementary Table 2, Supplementary Fig. 2A). Inferring others’ social emotions (social emotion > control) evoked significant activation in left supplementary motor area, superior frontal gyrus, inferior temporal gyrus, angular gyrus, precuneus, right cerebellum, and bilateral inferior and middle frontal gyrus, middle temporal gyrus and caudate (Supplementary Table 3, Supplementary Fig. 2B).

#### Effect of OT

**Fig. 3:**
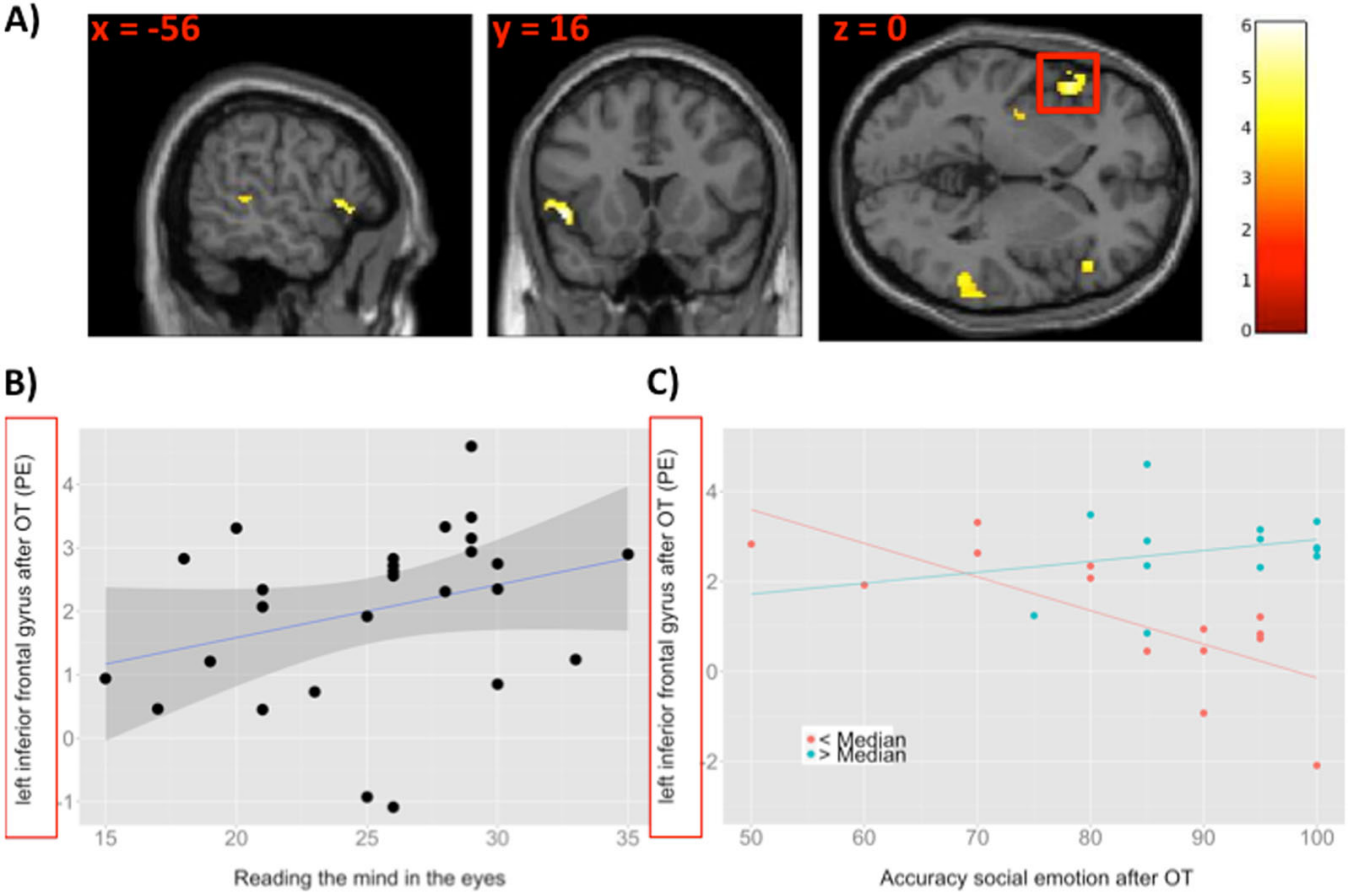
Imaging results. **a** Significant reduced activation in the left and right inferior frontal gyrus (IFG) during inferring others’ social emotion (social emotion > control) after OT compared with the placebo administration. Images are displayed at a cluster-forming threshold of *p*<0.001 uncorrected, with an extent threshold of 20 voxels. Colour bars indicate *t* values. **b** Significant correlation between left IFG activation after OT administration (i.e. parameter estimate, PE) during inferring others’ social emotion and baseline RMET performance (*r*=0.388, *p*=0.046). **c** Moderator effect of baseline RMET performance on the relationship between left IFG activation after OT administration (i.e. parameter estimate, PE) and task accuracy during inferring others’ social emotions after OT administration.

OT did not modulate brain activation during inferring others’ beliefs relative to placebo (Supplementary Table 4). However, during inferring others’ social emotions, OT significantly reduced activation in the left and right inferior frontal gyrus (Table 2, Fig. 3a). No significantly increased activation was found after OT relative to the placebo adiminstration during inferring others’ social emotions (Supplementary Table 5). Notably, the differences in left (*T* = 0.156, *p* = 0.879) and right (*T* = 0.873, *p* = 0.419) inferior frontal gyrus activation after OT relative to placebo did not differ between those individuals who received antidepressants (*n* = 8) or not.

**Table 2:** Treatments effects (placebo > OT) during inferring others’ social emotion (‘social emotion > control’).

Region, hemisphere	pFWE, cluster-level	Cluster size pFWE	*T* value	*Z* value	MNI coordinates (*x*/*y*/*z*)		
Inferior Frontal Gyrus, L	0.039	300	6.05	4.63	−48	18	−4
Inferior Frontal Gyrus, L			5.26	4.22	−30	36	−8
Inferior Frontal Gyrus, L			4.69	3.89	−56	16	2
Inferior Frontal Gyrus, R	0.042	294	5.52	4.36	48	26	−4
Inferior Frontal Gyrus, R			4.99	4.07	32	34	−8
Parahippocampus, R	0.563	71	4.68	3.88	30	−10	−28
Hippocampus, R			4.00	3.45	20	−10	−16
Middle temporal gyrus, R	0.099	216	4.65	3.87	58	−42	−4
Middle temporal gyrus, R			4.30	3.65	66	−36	−2
Inferior temporal gyrus, R			3.68	3.23	64	−44	−10
Postcentral gyrus, R	0.200	157	4.16	3.56	42	−22	34
Postcentral gyrus, R			3.93	3.40	36	−26	42
Postcentral gyrus, R			3.75	3.28	42	−28	58
Middle temporal gyrus, L	0.742	45	4.14	3.54	−60	−38	6
Calcarine, L	0.778	40	4.09	3.51	−26	−70	8
Calcarine, L			4.00	3.45	−24	−62	12
Putamen, L	0.854	29	4.08	3.50	−34	−10	−4
Parahippocampus, R	0.841	31	4.07	3.50	12	−22	−12
Cerebellum, L	0.899	22	3.95	3.42	−8	−42	−22

*FWE* family-wise error.

### Moderater effect of baseline social-emotional abilities on OT effects

We further tested whether left inferior frontal gyrus activation after OT administration during inferring others’ social emotions (social emotion > control) was related to baseline RMET scores. There was a significant positive correlation between baseline RMET scores and left inferior frontal gyrus activation after OT administration (*r* = 0.388, *p* = 0.046) (Fig. 3b). We further found that RMET score also moderated the relationship between left frontal gyrus activation during inferring others’ social emotion and task accuracy after OT administration, as indicatined by a significant interaction RMET × left inferior frontal gyrus (β=−3.53, *p* = 0.020). The moderator effect was also evident after median (median = 26) splitting the sample into high (*n* = 13) and low (*n* = 14) RMET performance (β=−20.05, *p* = 0.023). While task accuracy increased with decreasing activation in the left inferior frontal gyrus in CHR-P individual with low RMET scores (*r* = −0.578, *p* = 0.030), there was no significant relationship between task accuracy and left inferior frontal gyrus activation in CHR-P individual with high RMET scores (*r* = 0.088, *p* = 0.775) (Fig. 3c).

## Discussion

To the best of our knowledge, this is the first fMRI study to assess how acute OT administration modulated brain activation during inferring others’ beliefs and social emotions in CHR-P individuals. The first main finding is that OT did not affect brain responses during belief inference (cognitive empathy) but reduced activation in the bilateral inferior frontal gyrus during social emotion inference (emotional empathy). The second main finding is that neural activation in the left inferior frontal gyrus during social emotion inference after OT administration was modulated by baseline social-emotional abilities, which also moderated the relationship between brain activation and task performance. These findings can inform subsequent experimental therapeutics in this patient population.

The first finding of this study is that while OT did not modulate neural responses during belief inference it did reduce neural activation in the bilateral inferior frontal gyrus during social emotion inference. OT has been shown to enhance emotional but not cognitive empathy in healthy participants^25,33^, an effect that could be replicated across culture and sex^53^. In early psychosis patients, OT treatment did not enhance cognitive empathy but improved the ability to recognize emotions in schizophrenia patients^27^. We add to this line of evidence by showing that OT significantly decreased activation in the bilateral inferior frontal gyrus during emotional but not cognitive empathy in CHR-P subjects. The inferior frontal gyrus has been increasingly implicated in emotion recognition tasks such as identification of emotional intonation^54^, judgment of facial expressions^55^ and is an essential part of the neural mirror system, which is involved in a variety of important social behaviours, from imitation to emotional empathy^56^. Good imitation facilitates recognizing emotions in other people and thus emotional empathy^57^. Previous seminal work demonstrated that patients with inferior frontal gyrus lesions displayed extremely impaired emotional but not cognitive empathy^12^. More importantly, a previous study that used the same fMRI task in CHR-P found reduced activation in the bilateral inferior frontal gyrus when inferring others emotions but not beliefs relative to healthy controls^16^. The inferior frontal gyrus is a core target of the neurofunctional effects of OT^58^. OT may promote assimilation of novel emotional experiences into internal models^59^, which is supported by parts of the left inferior frontal gyrus^60^. Emotion perception is impaired in patients with schizophrenia^1^, CHR-P subjects^61^, offspring of parents with schizophrenia^62^ and is related to poor functional outcomes^1,61^. As a potential compensatory mechanism for this deficit, stronger inferior frontal gyrus activation during emotional perspective taking has been observed in CHR-P subjects compared with healthy controls^14,15^. Given that task performance during emotional empathy did not differ between OT and placebo in the current study, decreased inferior frontal gyrus activation after OT administration might indicate enhanced neural efficiency. Such an interpretation corresponds with a recent study showing that OT enhances neural efficiency of social perception as expressed by reduced event-related potential latencies^63^. Taken together, we might speculate that after receiving placebo, CHR-P subjects compensated their deficits in emotional perspective-taking by increasing activation in the inferior frontal gyrus compared with healthy individuals, as previously shown^14,15^, whereas OT may enhance neural efficiency in the inferior frontal gyrus by normalizing activation to the level of healthy persons.

The second main finding was of a positive relationship between baseline social-emotional abilities and left inferior frontal gyrus activation during social emotion inference (emotional empathy) after OT administration. Moreover, baseline social-emotional abilities as expressed by RMET performance moderated the relationship between OT-induced task performance and inferior frontal gyrus activation during emotional empathy. In particular, whereas inferior frontal gyrus activation decreased with increasing task performance in CHR-P subjects with low baseline social-emotional abilities, no such relationship was found in CHR-P subjects with high baseline social-emotional abilities. These findings suggest that OT-induced improvement in neural efficiency as expressed by decreased activation in the inferior frontal gyrus only took place in those CHR-P subjects with low baseline social-emotional abilities. In other words, CHR-P subjects with low social-emotional abilities needed less activation in the inferior frontal gyrus to achieve comparable task performance as after placebo administration. This finding resonates with previous reports demonstrating that OT selectively improved mind-reading as assessed with the RMET in those individuals with low emotional empathy^26^, high in alexithymia or high in maternal love withdrawal^34,64,65^. Although RMET performance has mostly been associated with cognitive empathy^26^, a recent study demonstrated that the RMET measures emotion recognition rather than theory of mind (i.e. cognitive empathy) ability^66^. Taken together, there is emerging evidence that OT influences emotional rather than cognitive processing and that its benefit depends on emotional empathy capacity.

There are several possible clinical implications. The dearth of preventive treatments that can effectively impact clinical outcomes—in particular social functioning deficits— can be partially explained by lack of clear aetiopathological mechanisms underlying the therapeutic effects of preventive agents. This study advances knowledge in this area by providing the first disease target-engagement evidence in CHR-P individuals. These neurophysiological results can inform the development of subsequent randomised controlled trials investigating OT’s effects on CHR-P symptoms and on clinical outcomes. Identifying novel therapeutic target for outcomes other than psychosis onset is a mainstream of future research in this area, considering that most CHR-P individuals who will not transition to psychosis will still display social functioning deficits at follow-up^67^.

The present study has limitations. No healthy control group was included in the current study. However, we have used a fMRI task which was already tested in CHR-P vs. healthy controls^16^, demonstrating significant alterations in key brain areas that are also implicated in this study. Due to sex differences in brain responses to OT during social interaction^68^, only male subjects were included. Another limitation is that this study did not collect extensive data on negative symptoms or neurocognition. Dose-response assessments are further required to test whether lower dosing might be sufficient for optimal target engagement, i.e. improving neural efficiency in the inferior frontal gyrus. While this study demonstrated that acute OT administration modulates key brain regions during inferring others’ social emotions, OT effects on functional outcomes in CHR-P subjects need to be tested in future longer-term clinical trials. Furthermore, in terms of the neurophysiological mechanisms by which OT has its effects, we previously reported that OT modulates cerebral (hippocampal) perfusion in CHR-P patients^35^, but did not appear to modulate regional concentrations of neurochemical metabolites^36^. The mechanisms by which OT increases neural efficiency in CHR-P individuals therefore warrants further research. A final limitation is that because of the small sample size we have been unable to stratify the heterogeneous CHR-P group across its three main subgroups^69^.

In conclusion, the present study provides the first evidence that acute OT administration modulates brain regions while inferring others’ social emotions in CHR-P subjects. Abnormal emotion perception persists over the course of the illness^70^ and is predictive for poor functional outcomes in CHR-P subjects^61^. This study may thus provide neural targets to develop novel treatments for socio-emotional deficits in CHR-P subjects with the aim to improve functional outcomes.

## Supplementary information

Supplementary Information
