# Supplementary material for: Acute oxytocin effects in inferring others’ beliefs and social emotions in people at clinical high risk for psychosis
Source: Transl Psychiatry. 2020 Jun 22;10:203. doi: 10.1038/s41398-020-00885-4 (PMC7308367; doi:10.1038/s41398-020-00885-4)
Supplement: Supplementary file 1 — Supplementary Information [file 41398_2020_885_MOESM1_ESM.docx]

**Supplementary material**

**Acute oxytocin effects in inferring others’ beliefs and social emotions in people at clinical high risk for psychosis**

André Schmidt^1^, Cathy Davies^2^, Yannis Paloyelis^3^, Nicholas Meyer^4^, Andrea De Micheli^2,5^, Valentina Ramella-Cravaro^2^, Umberto Provenzani^2,6^, Yuta Aoki^7,8,9^, Grazia Rutigliano^2^, Marco Cappucciati^2^, Dominic Oliver^2^, Silvia Murguia^10^, Fernando Zelaya^3^, Paul Allen^4,11^, Sukhi Shergill^4^, Paul Morrison^4^, Steve Williams^3^, David Taylor^12^, Stefan Borgwardt^1^, Hidenori Yamasue^7^, Philip McGuire ^4,5,13^, Paolo Fusar-Poli^2,4,6,13^

^1^ University of Basel, Department of Psychiatry (UPK), Basel, Switzerland; ^2^ Early Psychosis: Interventions and Clinical-detection (EPIC) lab, Department of Psychosis Studies, Institute of Psychiatry, Psychology & Neuroscience, King’s College London, London, UK; ^3^ Department of Neuroimaging, Institute of Psychiatry, Psychology & Neuroscience, King’s College London, London, UK; ^4^ Department of Psychosis Studies, Institute of Psychiatry, Psychology & Neuroscience, King's College London, London, UK; ^5^ National Institute for Health Research (NIHR) Biomedical Research Centre (BRC), South London and Maudsley NHS Foundation Trust, London, UK; ^6^ Department of Brain and Behavioural Sciences, University of Pavia, Pavia, Italy; ^7^ Department of Psychiatry, Hamamatsu University School of Medicine, Shizuoka, Japan; ^8^ Medical Institute of Developmental Disabilities Research, Showa University, Tokyo, Japan; ^9^ Department of Psychosocial Medicine, National Center for Child Health and Development, Tokyo, Japan; ^10^ Tower Hamlets Early Detection Service (THEDS), East London NHS Foundation Trust, London, UK; ^11^ Department of Psychology, University of Roehampton, London, UK; ^12^ Institute of Pharmaceutical Science, King's College London, London, UK; ^13^ Outreach and Support in South London (OASIS) Service, South London and Maudsley NHS Foundation Trust, London, UK;

**Supplementary Figure 1.** All 10 different stories presented during the task.

**
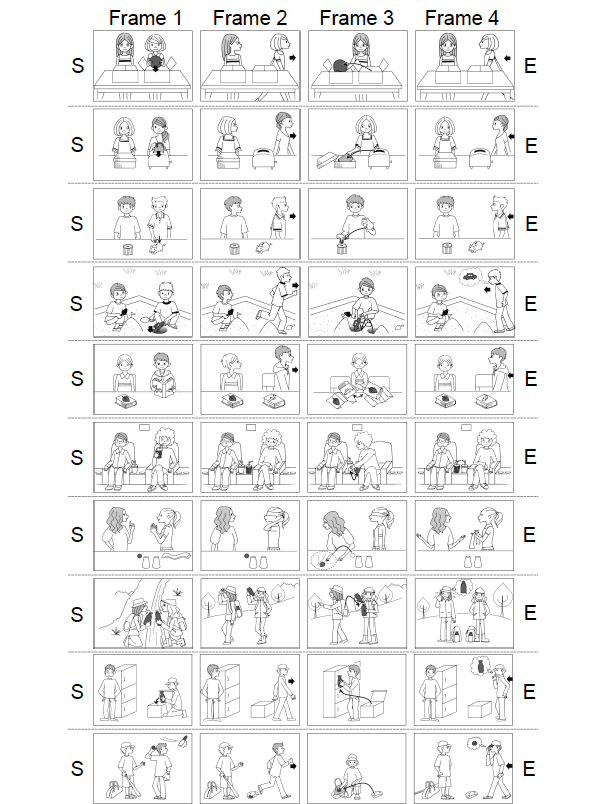
**

**Supplementary table 1.** Spearman correlations among performance measures within and between task conditions

|  | **PLA_control_RT** | **PLA_belief_RT** | **PLA_social_RT** | **OT_control_RT** | **OT_belief_RT** | **OT_social_RT** | **PLA_control_ACC** | **PLA_belief_ACC** | **PLA_social_ACC** | **OT_control_ACC** | **OT_belief_ACC** | **OT_social_ACC** |
| --- | --- | --- | --- | --- | --- | --- | --- | --- | --- | --- | --- | --- |
| **PLA_control_RT** | r=1.000 | r=,765^**^, p=0.000 | r=,678^**^, p=0.000 | r=,770^**^, p=0.000 | r=,501^**^, p=0.008 | r=,568^**^, p=0.002 | r=-0.338, p=0.085 | r=-,433^*^, p=0.024 | r=-0.089, p=0.660 | r=-,413^*^, p=0.032 | r=-0.343, p=0.080 | r=-0.259, p=0.193 |
| **PLA_belief_RT** | r=,765^**^, p=0.000 | r=1.000 | r=,782^**^, p=0.000 | r=,490^**^, p=0.009 | r=,397^*^, p=0.040 | r=0.328, p=0.094 | r=-,402^*^, p=0.038 | r=-,453^*^, p=0.018 | r=-0.199, p=0.320 | r=-0.265, p=0.181 | r=-0.154, p=0.444 | r=-0.254, p=0.201 |
| **PLA_social_RT** | r=,678^**^, p=0.000 | r=,782^**^, p=0.000 | r=1.000 | r=,574^**^, p=0.002 | r=,459^*^, p=0.016 | r=,427^*^, p=0.026 | r=-0.355, p=0.070 | r=-0.300, p=0.129 | r=-0.365, p=0.061 | r=-0.120, p=0.552 | r=0.002, p=0.991 | r=-0.136, p=0.500 |
| **OT_control_RT** | r=,770^**^, p=0.000 | r=,490^**^, p=0.009 | r=,574^**^, p=0.002 | r=1.000 | r=,787^**^, p=0.000 | r=,762^**^, p=0.000 | r=-0.182, p=0.364 | r=-0.224, p=0.261 | r=0.022, p=0.914 | r=-0.287, p=0.146 | r=-0.378, p=0.052 | r=-0.052, p=0.798 |
| **OT_belief_RT** | r=,501^**^, p=0.008 | r=,397^*^, p=0.040 | r=,459^*^, p=0.016 | r=,787^**^, p=0.000 | r=1.000 | r=,832^**^, p=0.000 | r=-0.155, p=0.441 | r=-0.028, p=0.890 | r=0.006, p=0.976 | r=-0.181, p=0.365 | r=-,409^*^, p=0.034 | r=-0.071, p=0.727 |
| **OT_social_RT** | r=,568^**^, p=0.002 | r=0.328, p=0.094 | r=,427^*^, p=0.026 | r=,762^**^, p=0.000 | r=,832^**^, p=0.000 | r=1.000 | r=-0.067, p=0.740 | r=0.134, p=0.505 | r=0.185, p=0.355 | r=-0.067, p=0.738 | r=-0.266, p=0.180 | r=0.027, p=0.895 |
| **PLA_control_ACC** | r=-0.338, p=0.085 | r=-,402^*^, p=0.038 | r=-0.355, p=0.070 | r=-0.182, p=0.364 | r=-0.155, p=0.441 | r=-0.067, p=0.740 | r=1.000 | r=,495^**^, p=0.009 | r=,432^*^, p=0.024 | r=,474^*^, p=0.012 | r=0.370, p=0.058 | r=0.094, p=0.640 |
| **PLA_belief_ACC** | r=-,433^*^, p=0.024 | r=-,453^*^, p=0.018 | r=-0.300, p=0.129 | r=-0.224, p=0.261 | r=-0.028, p=0.890 | r=0.134, p=0.505 | r=,495^**^, p=0.009 | r=1.000 | r=0.363, p=0.063 | r=,605^**^, p=0.001 | r=0.311, p=0.114 | r=0.162, p=0.419 |
| **PLA_social_ACC** | r=-0.089, p=0.660 | r=-0.199, p=0.320 | r=-0.365, p=0.061 | r=0.022, p=0.914 | r=0.006, p=0.976 | r=0.185, p=0.355 | r=,432^*^, p=0.024 | r=0.363, p=0.063 | r=1.000 | r=0.196, p=0.327 | r=0.231, p=0.246 | r=0.219, p=0.273 |
| **OT_control_ACC** | r=-,413^*^, p=0.032 | r=-0.265, p=0.181 | r=-0.120, p=0.552 | r=-0.287, p=0.146 | r=-0.181, p=0.365 | r=-0.067, p=0.738 | r=,474^*^, p=0.012 | r=,605^**^, p=0.001 | r=0.196, p=0.327 | r=1.000 | r=,699^**^, p=0.000 | r=0.316, p=0.108 |
| **OT_belief_ACC** | r=-0.343, p=0.080 | r=-0.154, p=0.444 | r=0.002, p=0.991 | r=-0.378, p=0.052 | r=-,409^*^, p=0.034 | r=-0.266, p=0.180 | r=0.370, p=0.058 | r=0.311, p=0.114 | r=0.231, p=0.246 | r=,699^**^, p=0.000 | r=1.000 | r=0.296, p=0.133 |
| **OT_social_ACC** | r=-0.259, p=0.193 | r=-0.254, p=0.201 | r=-0.136, p=0.500 | r=-0.052, p=0.798 | r=-0.071, p=0.727 | r=0.027, p=0.895 | r=0.094, p=0.640 | r=0.162, p=0.419 | r=0.219, p=0.273 | r=0.316, p=0.108 | r=0.296, p=0.133 | r=1.000 |

^*^Significant correlations; PLA, placebo; OT, oxytocin; RT, reaction time; ACC, accuracy

**Supplementary table 2.** Brain activation during inferring other’s beliefs (belief > control)

| **Region, Hemisphere** | **pFWE Cluster-level** | **Cluster size** | **T** | **Z** | **MNI coordinates (x/y/z)** | | |
| --- | --- | --- | --- | --- | --- | --- | --- |
| Precuneus, L | 0.000 | 334 | 8.55 | 5.79 | 2 | -62 | 38 |
| Angular gyrus, L |  |  | 8.35 | 5.72 | -46 | -60 | 32 |
| Inferior parietal gyrus; L |  |  | 7.51 | 5.38 | -40 | -56 | 52 |
| Inferior parietal gyrus; L |  |  | 7.51 | 5.38 | -46 | -52 | 38 |
| Middle frontal gyrus, L | 0.000 | 294 | 8.10 | 5.62 | -30 | 16 | 42 |
| Middle frontal gyrus, L |  |  | 6.93 | 5.13 | -36 | 10 | 50 |
| Middle temporal gyrus, R | 0.000 | 161 | 7.25 | 5.27 | 52 | -68 | 14 |
| Middle temporal gyrus, R |  |  | 7.18 | 5.24 | 56 | -64 | 22 |
| Inferior frontal gyrus, L | 0.000 | 109 | 6.43 | 4.90 | -54 | 22 | 26 |
| Inferior frontal gyrus, L |  |  | 6.32 | 4.84 | -42 | 22 | 26 |
| Inferior frontal gyrus, L |  |  | 5.80 | 4.58 | -32 | 22 | 20 |

FWE, family-wise error

**Supplementary table 3.** Brain activation during inferring other’s emotions (social emotion > control)

| **Region, Hemisphere** | **pFWE, Cluster-level** | **Cluster size** | **T** | **Z** | **MNI coordinates (x, y, z)** | | |
| --- | --- | --- | --- | --- | --- | --- | --- |
| Inferior frontal gyrus, L | 0.000 | 3088 | 14.20 | 7.36 | -56 | 24 | 16 |
| Inferior frontal gyrus, L |  |  | 12.18 | 6.90 | -48 | 42 | -2 |
| Inferior frontal gyrus, L |  |  | 11.84 | 6.81 | -46 | 36 | -14 |
| Middle temporal gyrus, R | 0.000 | 3731 | 13.95 | 7.31 | 56 | -56 | 16 |
| Middle temporal gyrus, R |  |  | 12.93 | 7.08 | 48 | -60 | 10 |
| Inferior temporal gyrus, R |  |  | 12.07 | 6.87 | 48 | -68 | -4 |
| Superior frontal gyrus, L | 0.000 | 3233 | 13.73 | 7.26 | -6 | 54 | 28 |
| Supplementary Motor Area, L |  |  | 13.42 | 7.19 | -8 | 18 | 58 |
| Superior frontal gyrus, L |  |  | 12.97 | 7.09 | -6 | 54 | 40 |
| Middle temporal gyrus, L | 0.000 | 4039 | 12.17 | 6.90 | -60 | -54 | 8 |
| Angular gyrus, L |  |  | 12.11 | 6.88 | -52 | -60 | 26 |
| Middle temporal gyrus, L |  |  | 12.07 | 6.87 | -48 | 14 | -34 |
| Inferior frontal gyrus, R | 0.000 | 979 | 10.84 | 6.54 | 56 | 26 | 2 |
| Inferior frontal gyrus, R |  |  | 9.99 | 6.29 | 46 | 30 | -10 |
| Inferior frontal gyrus, R |  |  | 7.28 | 5.29 | 56 | 24 | 20 |
| Precuneus, L | 0.000 | 781 | 10.09 | 6.32 | -2 | -60 | 32 |
| Cerebellum, R | 0.000 | 133 | 7.84 | 5.52 | 12 | -78 | -34 |
| Cerebellum, R |  |  | 6.83 | 5.08 | 28 | -78 | -36 |
| Cerebellum, R |  |  | 5.96 | 4.66 | 32 | -70 | -32 |
| Caudate, L | 0.000 | 246 | 7.37 | 5.32 | -14 | 4 | 12 |
| Caudate, L |  |  | 6.94 | 5.13 | -10 | 14 | 6 |
| Middle frontal gyrus, R | 0.000 | 136 | 6.86 | 5.10 | 50 | 24 | 40 |
| Middle frontal gyrus, R |  |  | 6.75 | 5.05 | 48 | 10 | 46 |
| Cerebellum, R | 0.003 | 27 | 6.67 | 5.01 | -18 | -76 | -36 |
| Caudate, R | 0.003 | 30 | 6.21 | 4.78 | 16 | 6 | 12 |

FWE, family-wise error

**Supplementary Figure 2.** Brain activation (effect of condition) during inferring other’s beliefs and social emotions after placebo


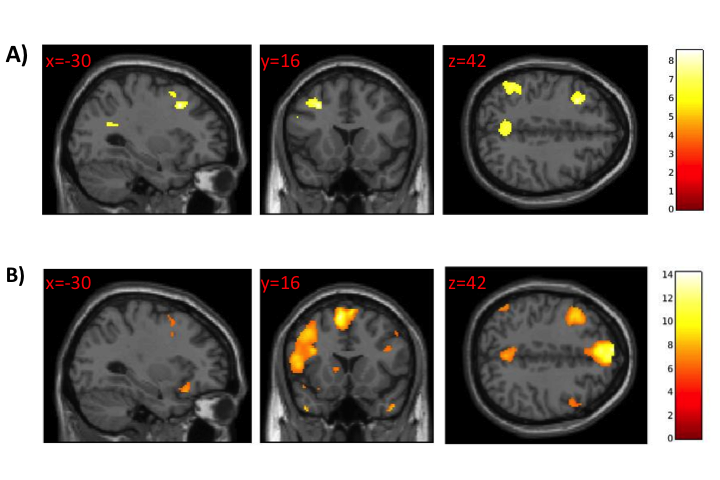


**A)** Belief > control and **B)** social emotion > control. Results are displayed at a threshold of p < 0.05 family-wise error (FWE) corrected across the whole brain. Colour bars indicate t values.

**Supplementary table 4.** Treatment effects during inferring other’s beliefs (belief > control)

| **Region, Hemisphere** | **pFWE, Cluster-level** | **Cluster size** | **T** | **Z** | **MNI coordinates (x, y, z)** | | |
| --- | --- | --- | --- | --- | --- | --- | --- |
| **PLA > OT** | | | | | | | |
| Superior frontal gyrus, R | 0.698 | 51 | 4.60 | 3.84 | 22 | -2 | 64 |
| Thalamus, R | 0.835 | 32 | 4.50 | 3.77 | 26 | -20 | 14 |
| Superior frontal gyrus, L | 0.876 | 26 | 4.00 | 3.45 | -18 | 8 | 48 |
| Insula, L | 0.727 | 47 | 3.96 | 3.42 | -36 | -8 | 2 |
| Supramarginal gyrus, L | 0.895 | 23 | 3.94 | 3.41 | -50 | -22 | 34 |
| Postcentral gyrus, L | 0.913 | 20 | 3.90 | 3.38 | -44 | -14 | 5 |
| **PLA < OT** | | | | | | | |
| Posterior cingulate cortex, L | 0.757 | 43 | 4.63 | 3.85 | -16 | -40 | 30 |
| Precuneus, R | 0.814 | 35 | 4.52 | 3.78 | 14 | -44 | 30 |
| Superior frontal gyrus, R | 0.807 | 36 | 4.41 | 3.71 | 14 | 64 | 24 |
| Superior frontal gyrus, R |  |  | 4.07 | 3.50 | 10 | 58 | 34 |

FWE, family-wise error

**Supplementary table 5.** Treatment effects (OT > placebo) during inferring others’ social emotion (social emotion > control)

| **Region, Hemisphere** | **pFWE, Cluster-level** | **Cluster size**  **pFWE** | **T value** | **Z value** | **MNI coordinates (x/y/z)** | | |
| --- | --- | --- | --- | --- | --- | --- | --- |
| Superior occipital lobe, L | 0.742 | 45 | 5.64 | 4.42 | -14 | -88 | 32 |
| Midcingulate cortex, L | 0.550 | 73 | 5.26 | 4.22 | -20 | 0 | 36 |
| Cuneus, R | 0.887 | 24 | 4.58 | 3.82 | 14 | -84 | 38 |
| Superior temporal pole, L | 0.905 | 21 | 4.17 | 3.56 | -48 | 10 | -20 |
| Caudate, L | 0.685 | 53 | 4.03 | 3.47 | -18 | -24 | 30 |
| Superior frontal gyrus, R | 0.911 | 20 | 3.66 | 3.22 | 34 | 56 | 20 |

FWE, family-wise error
